# Near real-time vegetation anomaly detection with MODIS NDVI: Timeliness vs. accuracy and effect of anomaly computation options

**DOI:** 10.1016/j.rse.2018.11.041

**Published:** 2019-02

**Authors:** Michele Meroni, Dominique Fasbender, Felix Rembold, Clement Atzberger, Anja Klisch

**Affiliations:** a European Commission, Joint Research Centre (JRC), Via E. Fermi 2749, I-21027 Ispra, VA, Italy; b Institute for Surveying, Remote Sensing and Land Information, University of Natural Resources and Life Sciences (BOKU), Vienna, Peter Jordan Straße 82, A-1190 Vienna, Austria

**Keywords:** Early warning, MODIS, NDVI, Anomalies, Near real-time estimation, Timeliness, Accuracy

## Abstract

For food crises early warning purposes, coarse spatial resolution NDVI data are widely used to monitor vegetation conditions in near real-time (NRT). Different types of NDVI anomalies are typically employed to assess the current state of crops and rangelands as compared to previous years. Timeliness and accuracy of such anomalies are critical factors to an effective monitoring. Temporal smoothing can efficiently reduce noise and cloud contamination in the time series of historical observations, where data points are available before and after each observation to be smoothed. With NRT data, smoothing methods are adapted to cope with the unbalanced availability of data before and after the most recent data points. These NRT approaches provide successive updates of the estimation of the same data point as more observations become available. Anomalies compare the current NDVI value with some statistics (e.g. indicators of central tendency and dispersion) extracted from the historical archive of observations. With multiple updates of the same datasets being available, two options can be selected to compute anomalies, i.e. using the same update level for the NRT data and the statistics or using the most reliable update for the latter. In this study we assess the accuracy of three commonly employed 1 km MODIS NDVI anomalies (standard scores, non-exceedance probability and vegetation condition index) with respect to (1) delay with which they become available and (2) option selected for their computation. We show that a large estimation error affects the earlier estimates and that this error is efficiently reduced in subsequent updates. In addition, with regards to the preferable option to compute anomalies, we empirically observe that it depends on the type of application (e.g. averaging anomalies value over an area of interest vs. detecting “drought” conditions by setting a threshold on the anomaly value) and the employed anomaly type. Finally, we map the spatial pattern in the magnitude of NRT anomaly estimation errors over the globe and relate it to average cloudiness.

## Highlights

• We compute three types of near real-time coarse resolution NDVI anomalies.
• Errors in near real-time anomaly estimation are assessed.
• We investigate the trade-off between timeliness and accuracy.
• We explore two options for the computation of near real-time anomalies
• The choice of the best option depends on the type of application and the employed anomaly type.

## 1 Introduction

The availability of near real-time (NRT) vegetation indexes (e.g. Normalized Difference Vegetation Index, NDVI) and biophysical variables (e.g. Fraction of Absorbed Photosynthetically Active Radiation, FAPAR; Leaf Area Index, LAI) is essential for the operational monitoring of vegetation conditions. In this framework, with NRT we refer to production delays in the order of few days, typically two or less. This type of NRT satellite observations are for example routinely analysed by international programs and agencies monitoring food security^1^ as they provide valuable information about crops and rangelands status (Brown, 2008; Brown and Brickley, 2012; Senay et al., 2014; Atzberger et al., 2016; Rembold et al., 2016). The effectiveness of this monitoring depends upon the availability of timely and accurate data.

Operational monitoring with coarse resolution remote sensing data in the reflected domain generally relies on so-called temporal composite products. In compositing, quality data are selected as the most reliable observation within a given temporal window, usually of a minimum of 10 or 16 days when the instrument acquires daily observations. Widely used datasets are for instance the maximum value NDVI composites (Holben, 1986) derived from atmospherically corrected daily observation of the Moderate Resolution Imaging Spectroradiometer (MODIS, 16-day composites) and Satellite Pour l'Observation de la Terre (SPOT) -VEGETATION/Proba-V (VGT/PV, 10-day composites). The resulting time series are nonetheless affected by residual cloud contamination, atmospheric variability, and bi-directional effects (Cihlar et al., 1997). Temporal smoothing is then applied to these time series to reduce the residual noise (Goward et al., 1991; Chen et al., 2004) and to fill possibly remaining data gaps (Weiss et al., 2014). Data gaps occur for example as a result of prolonged cloud coverage. Various smoothing algorithms have been proposed in the literature, for recent review and comparison see for example Shao et al. (2016) and Kandasamy et al. (2013).

Unlike the off-line smoothing of historical time series when observations are available before and after each temporal data point, NRT filtering requires the projection to the current time from the past observations only (Sedano et al., 2014). The NRT estimation made at time t_0_ is referred to as consolidation stage 0 (Klisch and Atzberger, 2016; Verger et al., 2014). At the following time step (t_0_ + 1), with additional observations potentially becoming available, a more reliable estimation is produced for t_0_. This is referred to as consolidation stage 1. Hence, as time progresses, the original estimation made for t_0_ is successively updated by consolidation stages of increasing order (and reliability) until stage *n*, produced at t_0+*n*_, the time at which the increase of quality resulting from the consideration of an additional data point after t_0_ is considered marginal. Obviously, during this process, also new estimations at consolidation stages 0 to *n* are produced for time t_0+1_ to t_0+*n*_. The decision of how many stages are calculated (*n*) is product specific. For instance, four stages are produced by the Copernicus Global Land for biophysical 1 km products building on SPOT-VEGETATION/Proba-V data (Smets et al., 2017), while six stages are computed by the University of Natural Resources and Life Sciences (BOKU) for NDVI building on MODIS data (Klisch and Atzberger, 2016).

The evaluation of the quality of off-line smoothing has received considerable attention in the literature while the quality of NRT products has been far less investigated. Brown et al. (2015) studied the quality of NDVI “expedited” eMODIS product compared to the standard MODIS product while Xiao et al. (2011), Verger et al. (2014) and Kandasamy et al. (2017) focussed on the quality of NRT LAI estimates.

For the first time, in this study we focus on the quality of the anomaly indicators derived from NRT NDVI data. Anomaly maps of NDVI are routinely used to detect anomalous crop and rangeland development compared to what can be assumed to be the average or “normal” situation. For this purpose, statistics of central tendency and dispersion computed on the historical archive (HIS) of the NDVI can be used (Eerens et al., 2014; Rembold et al., 2013). The full set of available past observations or a subset of it (e.g. the previous 5 years) can be consider to define HIS. To provide information without delay, NDVI anomalies are computed comparing the recent unconsolidated (i.e. subjected to updates) NRT NDVI value with the distribution of NDVI values observed in the past years at the same time of the year. To estimate the quality of such unconsolidated NRT anomalies we compare them with the reference provided by the fully consolidated time series, i.e. obtained through off-line smoothing of the original observations. These “reference anomalies” can be computed retrospectively, but they are obviously not available in NRT. It is noted that analysing the error structure of anomalies instead of that of the original NDVI is important because relatively small errors in NDVI data may be amplified by the anomaly computation, especially in areas characterised by small inter-annual variability. Therefore, errors that may be considered negligible for other type of applications relying on original NDVI data may become relevant for applications using anomalies (e.g. drought detection and early warning).

The objectives of this study are to quantify the error of NRT anomaly estimation and to explore the error magnitude with respect to (1) delay with which they become available and (2) different options selected for their computation. These options refer to the use of different consolidation stage selected for the HIS data set, on which the statistics used in the computation of the anomalies are extracted. The relation between the estimation errors and cloudiness, potentially reducing the number of available observations and thus making the NRT filtering challenging, is investigated to provide a possible explanation of observed global spatial pattern of errors.

In parallel, we also analyse the effect of estimation errors in two secondary products derived from anomalies that are often used by agricultural analysists. First, the classification of the anomaly values into a few classes separating normal, above or below normal, and extreme conditions. This classification is typically performed to improve the readability of anomaly maps. Second, the detection of (very) large negative anomalies. Indeed, NDVI anomalies can be used to identify infrequent and negative events such as droughts by setting a threshold on the anomaly to discriminate severe negative conditions (e.g. Rojas et al., 2011; Sepulcre-Canto et al., 2012; Rembold et al., 2018). In this way, the continuous anomaly variable is discretised into a dichotomous (yes/no) variable representing the occurrence of the extreme event. In doing so, we are interested in detecting a specific type of negative and infrequent event. As the accuracy of such secondary products is not necessarily linked to the overall error of anomaly estimation, the quality of NRT estimation of such products is treated separately. We report error statistics calculated at the global scale and, as a case study example of regional drought monitoring, we show the temporal evolution of the onset of the late 2010 drought in the Horn of Africa as a function of consolidation stage.

## 2 Data

We use data from BOKU's MODIS data processing chain (Klisch and Atzberger, 2016). The data are operationally used by Kenya's National Drought Management Agency (NDMA) (https://ivfl-arc.boku.ac.at/kenya/map/) as well as for the JRC early warning system Anomaly Hotspots of Agricultural Production (ASAP, https://mars.jrc.ec.europa.eu/asap/, Rembold et al., 2018). The processing chain builds on MOD13A2 and MYD13A2 V006 16-day NDVI composites at 1 km resolution from the Terra and Aqua MODIS satellites. Although the two instruments have different overpass times, data from both instruments are combined to take advantage of more frequent (valid) observations. The NASA Land, Atmosphere Near real-time Capability for EOS (LANCE) system also provides NRT MODIS data with reduced latency compared to NASA LP DAAC (Land Processes Distributed Active Archive Center) used. However, LANCE data are only provided as rolling archive while the analysis described here requires the availability of the full historical datasets at all consolidation stages.

A total of 294 MODIS tiles cover the globe and are obtained through the online Data Pool at the NASA LP DAAC. Tiles are mosaicked and re-projected to geographic coordinates (datum WGS84, EPSG 4326) with a spatial resolution of approximately 1 km (1°/112) using nearest neighbour resampling.

The entire time series (from 2002) is first smoothed off-line using the Whittaker smoother (Atzberger and Eilers, 2011; Eilers, 2003). The smoother is employed here to smooth and interpolate the data in the historical archive to daily NDVI values. The Whittaker smoother balances two conflicting requirements (Eilers, 2003): i) fidelity to the data, and ii) smoothness of the resulting curve. The smoother attempts to both closely fit a curve to the raw data, but it is penalized if subsequent smoothed points vary too much (i.e. the fitting curve is not smooth). The smoothing parameter λ controls the balance between the two requirements (Eilers, 2003). The smaller λ, the more closely the smoothed curve follows the original observations, and vice versa. The appropriate values of λ for the smoothing of the historical archive and NRT data were identified through a trial and error process involving the visual inspection of a large number of sample points (observations and fitted curves) from different continents and environments.

The smoothing takes into account the quality of the observations according to the MODIS VI Quality Assessment Science Data Set (QA SDS) (Didan et al., 2015) and the actual acquisition day for each pixel. The weights assigned to the MODIS observations based on the QA SDS are reported in Klisch and Atzberger (2016). A smoothing parameter λ of 3000 is applied with three iterations to best fit the upper envelope of the NDVI observations similar to the procedure described in Beck et al. (2006). From the output of daily NDVI time series, only 10-day composites images are stored. The 10-day composites have a fixed date for the projection corresponding to the end-point of the respective dekad (i.e. day 1–10, 11–20, and 21–last day of the month).

NRT data are produced at the end of each dekad based on the data that are available at that time including the previous 190 days. It is noted that data availability is highly variable. NRT data production is referred to as filtering in contrast to the above described smoothing, following the definitions of Sedano et al. (2014). During filtering, a smoothing parameter λ of 1000 is used, reflecting the shorter time series compared to the off-line smoothing and providing the necessary flexibility of the smoothed curve. Filtered data are constrained by limiting the NDVI change between consecutive dekads according to historical statistics of the off-line smoothed data. Filtered unconstrained data refer to the NRT smoothed data produced without application of the previous constrain. This product is not publicly distributed and used in this study for explanation purposes only.

When a new NRT data point is produced, the estimates of the previous four dekads are also updated. As a result, NDVI dekadal images are produced at five consolidation stages (C0 to C4) with stage C4 coming with a 4 dekads delay and being the most reliable NRT filtered product. Additionally, a final and fully off-line smoothed product is produced (CF) with 3 months delay and serves in the present study as reference product. For NRT data production before the current date, hindcasting is used (e.g. simulating the incomplete availability of data as experienced in NRT operations) to produce the HIS archives of stages C0 to C4.

A global map of average annual cloud fraction of Wilson and Jetz (2016) is used in an attempt to provide an interpretation of the observed spatial error pattern. Cloud cover at 1 km resolution is derived from 15 years (2000–2014) of MODIS twice-daily observations making use of product MOD09GA and MYD09GA cloud flags. Data were downloaded from http://www.earthenv.org/cloud.

The analysis is performed at the global level for a 14 years period (2003–2016, for a total of 504 temporal samples per pixel). The global images are systematically spatially subsampled by selecting the central pixel in each non-overlapping window of 21 by 21 pixels. This subsample is representative for the global patterns of vegetation and considerably reduces processing time (Toté et al., 2017). Subsampling is not applied for the case study describing the drought onset in the Horn of Africa, for which we use full resolution data.

To focus on vegetation anomalies, error statistics are retrieved for all global vegetated land surfaces during their average growing season period. Vegetated areas and timing of the growing season are defined by the land surface phenology computed on the mean annual profile of the CF time series with the SPIRITS software (Eerens et al., 2014; Rembold et al., 2015). The software uses an approach based on thresholds on the green-up and decay phases as described in White et al. (1997). The following parameters are retrieved per pixel: number of growing season per year (i.e. one or two); start of season (SOS, occurring at the time at which NDVI grows above the 25% the ascending amplitude of the seasonal profile); and end of the season (EOS, when NDVI drops below 35% of the descending amplitude). SOS and EOS values are then used to define the growing season period that can be either single or double.

## 3 Methods

**Fig. 1:**
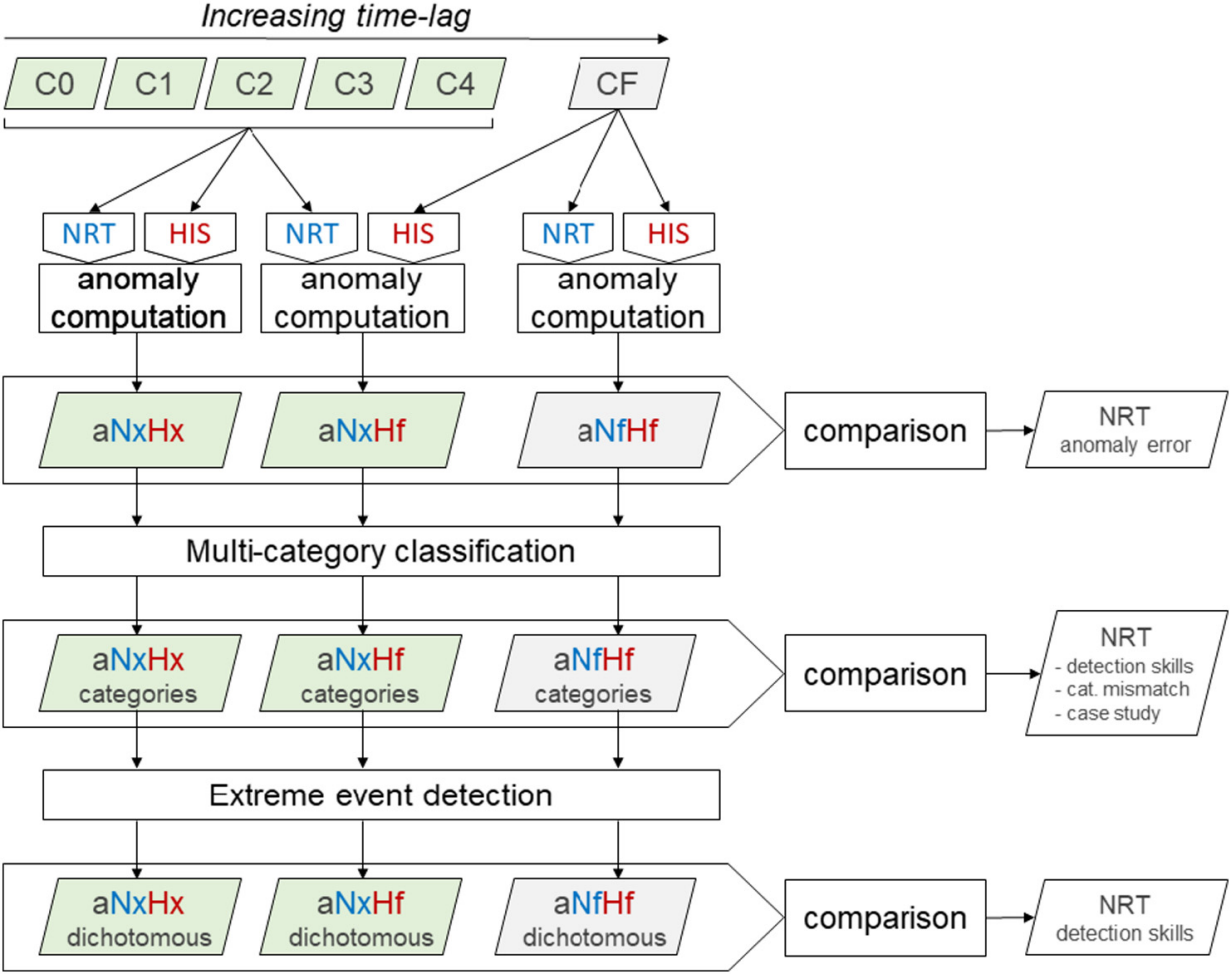
Flowchart summarising the main steps of the analysis. Green and grey shaded boxes represent near real-time (consolidation stages C0 to C4) and reference (CF) products, respectively. NRT (near real-time) and HIS (historical archive on which statistics are computed) are the input data for the anomaly computation. For the *a*N*x*H*y* nomenclature of anomalies see Table 1. (For interpretation of the references to colour in this figure legend, the reader is referred to the web version of this article.)

An overview of the analysis is provided in Fig. 1. Starting from the various consolidation stages (C0 to C4 produced in NRT, CF produced off-line) we compute various type of NRT and reference anomalies. The NRT anomalies are produced with the two type of computation options describe in Section 3.1. The errors in NRT anomaly are then evaluated by comparing NRT estimations with the reference value computed on the final stage CF (Section 3.2.1). The relation between such errors and cloudiness is explored (Section 3.3). Anomalies are then reclassified into few categories of interest (Section 3.2.2) and the mismatch between NRT anomaly categories and the corresponding reference categories is determined. Finally, by setting a threshold on the anomalies we test the NRT errors in detecting extreme (i.e. infrequent and negative) events (Section 3.2.3).

### 3.1 Computation of anomalies

Among the plethora of existing anomalies, we restrict the analysis to three commonly used types: i) standard score (z-score, Eq. (1)); ii) non-exceedance probability (NEP, also referred to as the percentile rank or Vegetation Productivity Index, VPI, Eq. (2)); and, iii) the Vegetation Condition Index (VCI, Eq. (3)). All anomalies are computed at the original dekadal time step, i.e. no temporal aggregation to longer periods is performed.

(1) $$Z_{i}=\left({\text{NDVI}}_{i}-{\text{NDVI}}_{\mathrm{mean},i}\right)/{\text{NDVI}}_{\mathrm{sd},i}$$

(2) $${\mathrm{NEP}}_{i}=\mathrm{rank}\left({\text{NDVI}}_{i}\right)/\left(n+1\right)*100$$

(3) $${\mathrm{VCI}}_{i}=\left({\text{NDVI}}_{i}-{\text{NDVI}}_{\min,i}\right)/\left({\text{NDVI}}_{\max,i}-{\text{NDVI}}_{\min,i}\right)*100$$

where NDVI_*i*_ is the NDVI at dekad *i* (*i* = 1, …, 36); NDVI_*mean,i*_, NDVI_*sd,i*_, NDVI_*min,i*_, and NDVI_*max,i*_, are the statistics extracted from the historical record of observations at dekad *i*: the mean value, the standard deviation, the minimum and the maximum values, respectively. The unbiased definition of NEP is used, dividing the rank (assigned in ascending order) of the observed NDVI within its historical distribution by the total number of observations (n) plus one.

All above anomalies are “normalized” anomalies in the sense that the data point is compared with some measure of dispersion in the observed distribution (i.e. not only with a measure of central tendency). In this way, NDVI observations at different locations and at different times can be compared in terms of how extreme they are. Standard score assumes normal distribution to normalize the NDVI. VCI (Kogan, 1995) uses the range of past observations to locate the value of an observation. Both anomaly types have been extensively used for drought detection (e.g. Kogan, 1997; Meroni et al., 2014; Rojas et al., 2011; Peters et al., 2002). NEP can be considered a non-parametric robust version of the standard score. In fact, under the assumption of normality of the data, standard score can be translated into a probability of non-exceedance.

In NRT operations, filtered products (consolidation stages C0 to C4) are used as NDVI_*i*_ in Eqs. (1), (2), (3), more or less delaying the provision of the anomaly indicators (from C0 being immediately available to C4 available after four dekads). The statistics used to compute the anomaly can potentially be computed on the historical archive of any of the six consolidation stages (C0 to CF). The method choice is not trivial. On the one hand, it appears appropriate to match the consolidation stage of the historical archive to that of the NRT product. In this case, one scales the NRT NDVI estimation with the statistics derived from its historical distribution. Bias and heteroscedasticity between NRT and reference data distributions should be accounted for in this way, potentially resulting in a NRT estimation closer to the reference. On the other hand, as we aim at estimating the anomaly as fully computed with the CF stage, it is also plausible to use the most reliable stage (i.e. CF) for the computation of the statistics. In this way, one makes use of reliable statistics to compute the anomalies. This option appears potentially valid for VCI in particular. In fact, VCI is computed using the minimum and maximum NDVI values at each dekad (Eq. (3)), thought to represent the range of climatic NDVI variation for a given location and time of the year (Unganai and Kogan, 1998). Outliers might be present in the first unconsolidated stages and progressively reduced with later stages. As sample extrema (i.e. minimum and maximum) are sensitive to outliers, their estimation from the reference stage CF might be more reliable.

There is probably a trade-off between sensitivity to outliers of the various statistics and theoretical consistency of the normalization. While the minimization of the outliers effect would lead to the use of the CF stage for the computation of the statistics, the theoretical consistency would lead to the use of the consolidation stage used in the NRT computation of the anomaly. We explore this trade-off by comparing the effect of the different computation options on the non-robust (Z, VCI) and robust (NEP) anomalies.

In practice, agencies with an interest in NRT monitoring generally use the statistics derived from the final and consolidated product (examples: FAO Agriculture Stress Index System, JRC MARS Crop Yield Forecasting System and ASAP early warning system, FEWSNET, WFP VAM). One notable exception is Kenya's National Drought Management Authority. The agency relies on BOKU University MODIS NDVI data and uses NRT and statistics extracted from the same consolidation stage. A practical advantage of using statistics from the final product is that the statistics can be computed once and then used with all consolidation stages. In addition, intermediate consolidation stages can be overwritten when more reliable stages are computed. On the contrary, pairing the consolidation stage of NRT and statistics requires storing multiple versions of the archive and its statistics.

For clarity we can define an anomaly as *a*N*x*H*y*, where *a* is the type of anomaly (*a* being z, v, and n for z-score, VCI and NEP, respectively), *x* is the consolidation stage of the NRT observation (N), and *y* is the consolidation stage of the historical archive (H) used to extract the required statistics. With this formalism, Table 1 lists the anomalies with different N*x*H*y* combination options considered in this analysis.

**Table 1:** Labelling of anomalies obtained using different consolidation stages. N*x*H*y* indicates an anomaly obtained with consolidation stage *x* for the NRT value and *y* for the historical statistics.

N*x*H*y* anomaly label	Stage *x* of NRT data	Stage *y* for the computation of statistics	Description
NfHf	f	f	Reference final anomaly
N0Hf, N1Hf, …, N4Hf	0 to 4	f	NRT data combined with statistics extracted from the reference
N0H0, N1H1, …, N4H4	0 to 4	0 to 4	NRT data combined with corresponding statistics extracted from the same consolidation stage

To mimic NRT operation we proceed as follows. When computing the anomaly for dekad *i* and year *j*, the NDVI observation (*i*,*j*) is included in the historical archive for the NRT anomalies of type N*x*H*x*, thus the anomalies computed using the same consolidation stage *x* for both NRT and statistics (i.e. N0H0, …, N4H4). For anomalies of type N*x*Hf (NRT stage *x* and statistics from final stage), the observation cannot be included in the historical archive as the CF stage would not be available in NRT. As a consequence, when NDVI_*i*_ value is not present in the historical distribution, NEP_*i*_ is computed by linear interpolation.

It is noted that the historical archive considered and the derived statistics refer in all cases to the whole 2003–2016 period. This would not have been the case in NRT operation in year *j* (with *j* < 2016), where only the period 2003–*j* would have been available. However, it appears appropriate to use the full period in order to study the accuracy of the two normalization options. In this way, any dekad during the period is treated identically and as if it was the last available, neglecting the effect of the time series length on the anomaly computation that is not of interest in the present study.

### 3.2 Computation of anomaly estimation error

The reference anomalies that we aim to estimate in NRT operations are those constructed with fully smoothed data (*a*NfHf). Therefore, all the error measures described below are based on the comparison between this reference and the anomaly estimation in NRT. Three types of error measures are identified to highlight the impact of estimation error on different types of anomaly use:

• Basic error statistics for anomalies;
• Error measures for multi-category anomaly classes;
• Error measures for dichotomous variables.

#### 3.2.1 Basic error statistics for anomalies

Error statistics are directly computed on anomaly values: i) the mean absolute error (MAE), being the absolute difference between the NRT anomaly estimate N*x*H*y* and the corresponding reference anomaly NfHf; and ii) the mean error (ME) to detect possible bias. These types of error should be typically minimized when computing spatial averages of the anomaly (e.g. administrative level values).

#### 3.2.2 Error measures for multi-category anomaly classes

To improve the readability of anomaly maps and to highlight major spatial patterns, anomaly values are often reclassified into a few classes separating normal, above or below normal, and extreme conditions. The number of classes and the thresholds employed are somehow subjective and vary among users. Here we use the setting proposed by the World Meteorological Organization for the Standardized Precipitation Index (a standardized index computed on cumulative rainfall; World Meteorological Organization, 2012) to classify z-score and NEP anomalies. For the latter we exploit the direct link between standard score and probability. To classify VCI anomalies we use the settings proposed by Klisch and Atzberger (2016) (Table 2). It is noted that the VCI classification is not symmetric around the central value, in contrast to the WMO classification.

**Table 2:** Classification of anomaly values into anomaly classes.

z-score		NEP		VCI	
Value	Class	Value	Class	Value	Class
x ≥ 2	Extremely good	x ≥ 97.7	Very to extr. good^a^	x ≥ 50	Better than average
1.5 ≤ x < 2	Very good	93.3 ≤ x < 97.7			
1 ≤ x < 1.5	Moderately good	84.1 ≤ x < 93.3	Moderately good		
−1 < x < 1	Near normal	15.9 < x < 84.1	Near normal	35 ≤ x < 50	Normal conditions
−1.5 < x ≤ −1	Moderately bad	6.7 < x ≤ 15.9	Moderately bad	20 ≤ x < 35	Moderate anomaly
−2 < x ≤ −1.5	Very bad	2.3 < x ≤ 6.7	Very to extr. bad^a^	10 ≤ x < 20	Severe anomaly
x ≤ −2	Extremely bad	x ≤ 2.3		x < 10	Extreme anomaly

a Note that classes “Extremely bad” and “Extremely good” are not present in NEP (having a minimum and maximum of 6.67% and 93.33% according to Eq. (3) with n = 14). Therefore, such classes are merged with the nearest ones.

Building on such a classification into multi-category anomaly classes, we compute the second type of error measure. The overall accuracy of *a*N*x*H*y* in mapping the reference classes of *a*NfHf informs us to what extent the NRT anomaly class distribution matches the reference one. As the overall accuracy is heavily influenced by the most represented classes (here the central classes representing normal situation), we focus on the Heidke skill score (HSS, equivalent to KHAT) as a measure of classification accuracy. HSS values are then interpreted following Congalton and Green (2008) and Landis and Koch (1977), thus dividing the possible ranges for HSS into three groups: a value >0.80 indicates strong agreement; a value between 0.40 and 0.80 indicates moderate agreement; and a value below 0.40 indicates poor agreement.

Despite being informative about classification accuracy, HSS equally penalizes all the possible misclassifications. That is, a NRT estimation “extremely bad” vs. a reference “extremely good” is weighted equally to a misclassification “moderately bad” vs. “”very bad” while the two misclassifications have obviously a different practical importance. To stress the differences between anomalies from an analyst's point of view we follow the approach of Meroni et al. (2016) and evaluate the agreement between the anomaly classes of Table 2 by computing the occurrence of the concordance classes reported in Table 3.

**Table 3:** Ranked concordance between anomaly classes of N*x*H*y* and NfHf.

Concordance class	Condition on the two anomalies
Agreement	Same class of anomaly
Minor mismatch	Same sign of the anomaly (above or below normal) but different magnitude
Mismatch	One is “normal” and the other moderately above or below normal
Serious mismatch	One is “normal” and the other largely above or below normal
Unacceptable mismatch	Opposite sign of the anomaly (above vs. below normal, any magnitude)

Compared to MAE and ME that focus on the difference of between two anomalies disregarding their values, the error measure based on the multi-category anomaly classes provides a direct indication of the severity of the error the analyst would face in interpreting the anomaly maps. Nevertheless, it is noted that the results of such analysis are dependent on the definition of the anomaly classes. That is, changing the thresholds in the definitions of the classes can potentially increase/decrease the agreement. The mismatch between anomalies is further illustrated qualitatively by showing temporal evolution of the onset of the late 2010 drought in the Horn of Africa as a function of consolidation stage (Fig. 3).

#### 3.2.3 Error measures for dichotomous variables

Finally, anomalies are often used to identify infrequent and negative events of poor vegetation development by setting a threshold value below which the negative event is assumed to have happened. This is a special case of the classification of the anomaly into n categories described above for which n = 2. In this way, the anomalies are translated into a dichotomous variable (yes/no of the “unfavourable” event) and can be used for example to compute the area subjected to drought (Rembold et al., 2018; Rojas et al., 2011) and, in case of Kenya NDMA, to provide objective triggers for releasing disaster contingency funds (Klisch and Atzberger, 2016).

For z-score we select the threshold of −1 SD used by WMO to classify the Standardized Precipitation Index (World Meteorological Organization, 2012) as “moderately” to “extremely dry”. Under the assumption of normal distribution, 15.9% of observations falls below this threshold. The same percentage is used as threshold for NEP. For VCI we select the 35% threshold used by Klisch and Atzberger (2016) to classify “moderate” to “extreme drought”. As VCI does not assume any particular distribution, no percentage can be a priori specified of how many observations fall below this threshold.

When comparing the NRT dichotomous estimates with the final reference, the following four combinations are possible: i) event estimated and occurred (hit); ii) event not estimated to occur, but occurred (miss); iii) event estimated to occur, but did not occur (false alarm); and, iv) event not estimated to occur and did not occur (correct negative). With this information, the third type of error measures are computed as statistics typically used in the verification of meteorological forecasts (Jolliffe and Stephenson, 2012) (Table 4).

**Table 4:** Statistics derived on the dichotomous classification of the anomalies (yes/no of the event “moderately bad or worse”). n is the total number of observations. h, m, fa, and cn stand for: hits, misses, false alarms, correct negatives.

Statistic	Equation	Range	Meaning
Bias	(h + fa) / (h + m) ∗ 100	[0–Inf]	Ratio between estimated and observed occurrence of the event. 100 is unbiased
Detection rate	h / (h + m) ∗ 100	[0−100]	Percentage of observed events that were correctly estimated
False alarm rate	fa / (h + fa) ∗ 100	[0–100]	Percentage of predicted events that eventually were not observed
Heidke skill score (HSS)	((h + cn) − ecr) / (n − ecr) where ecr = 1 / n ∗ ((h + m) ∗ (h + fa) + (cn + m) ∗ (cn + fa))	[−1:1]	Accuracy relative to that of random chance. 1 is perfect, 0 is no skill

### 3.3 Relation between anomaly estimation error and cloudiness

The per-pixel MAE of anomaly estimation for selected anomaly type and strategy is mapped to show existing globally spatial patterns. These patterns are then compared to those of mean annual cloud cover, considered as a proxy of the frequency of data gaps and potential atmospheric effect on observed NDVI (Eberhardt et al., 2016). Qualitative visual comparison and linear regression analysis between errors and cloudiness is performed.

## 4 Results and discussion

### 4.1 NRT anomaly estimation

**Fig. 2:**
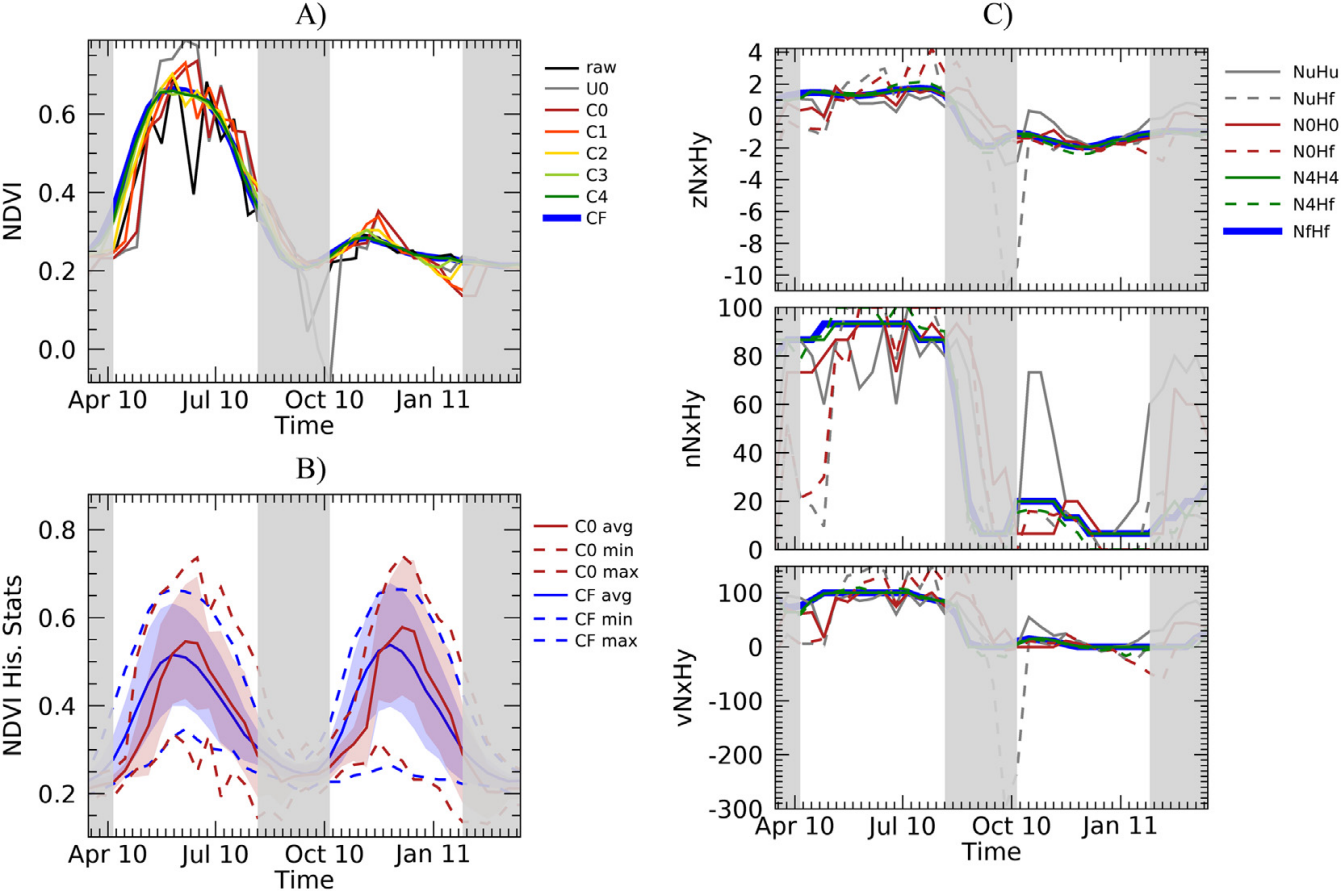
Temporal profiles over the period 10/03/2010–10/03/2011 for a crop pixel in Somalia (2.6786 N 44.1071 E). Grey vertical bands indicate times outside the average growing season periods. A): NDVI profiles. Raw data (raw), unconstrained smoothed data at stage 0 (U0), constrained filtered data at consolidation stages 0 to final (C0 to CF). B): NDVI historical average, minimum and maximum for stages 0 and F (C0, CF); shaded red and blue areas refer to average ± 1 SD. C) N*x*H*x* and N*x*Hf computation options for z-score, NEP and VCI anomalies computed with: unconstrained smoothed NDVI at stage 0 (U0), constrained filtered data at consolidation stages 0, 4 and final (C0, C4, and CF). (For interpretation of the references to colour in this figure legend, the reader is referred to the web version of this article.)

As an example, Fig. 2A shows the temporal profile of the raw NDVI observations, the filtered unconstrained NDVI at consolidation stage 0 (U0), and the filtered and constrained NDVI at each consolidation stage (C0 to C4 and CF). The temporal profiles refer to a crop pixel located in Somalia and showing bimodality (i.e. two growing seasons per year). The profiles cover a period of one solar year and two successive growing seasons. The very poor seasonal development of the first season of 2011 is clearly visible and was caused by a severe drought (Meroni et al., 2014). The drought started in late 2010 because of lack of rainfall during October–December (short rainfall season) that continued through March–May 2011 (long rainfall) and was attributed to La Niña conditions (Dutra et al., 2013).

Fig. 2A shows qualitatively how the agreement between the final and fully consolidated NDVI product (CF) and the NRT stages (C0 to 4) increases with higher consolidation levels. Fig. 2A also reports the raw NDVI observations and the unconstrained estimation at stage 0 (U0). Raw observations are plotted as a function of the actual day of acquisition. However, it is noted that they may not have been available at that time for the filtering process as the NDVI observations become available after the end of the 16-day MODIS temporal compositing period. The variability of the unconstrained estimation (i.e. pure NRT filtering without constrains on temporal evolution derived for historical archive) shows how NRT unconstrained filtering may lead to very high or low values, particularly at times of the year where rapid NDVI changes take place and/or many missing or unreliable inputs occur.

Fig. 2B shows the statistics of the NDVI historical archive used to compute the anomalies. A temporal shift between the first level of consolidation and the final one appears to be present. The results of the various computation options (i.e. N*x*Hf vs. N*x*H*x*) for three anomaly types are reported in Fig. 2C and compared with the reference profiles *a*NfHf. Only filtered consolidation stages 0 and 4 are reported to ensure readability of the graphic. The computation option N*x*H*x*, where the NRT observation at consolidation stage *x* is normalized using the historical data at the same consolidation stage, appears to be closer to the reference profile.

**Fig. 3:**
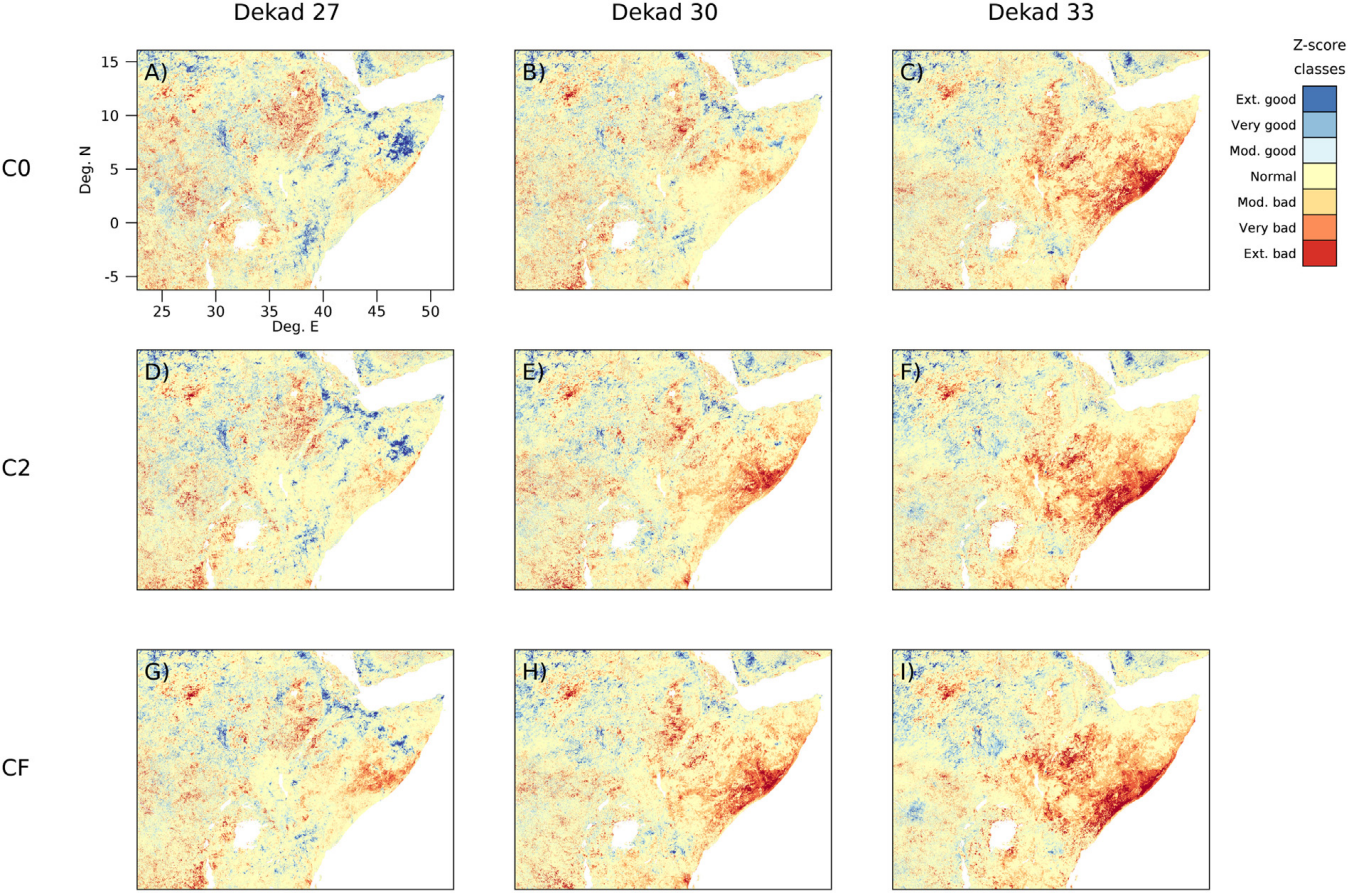
Maps of z-score anomaly classes over the Horn of Africa for different timings (columns) and consolidation stages (rows). Classes are defined according to Table 2. Time refers to dekad 27 (21–30 Sept.), 30 (21–31 Oct.), and 33 (21–30 Nov.) of year 2010. Consolidation stages are C0 (A–C), C2 (D–F) and reference CF (G–I). Z-score of stages C0 and C2 was computed using the N*x*H*x* computation option. All anomalies are computed on full resolution imagery (i.e. no subsampling).

As an example of resulting anomaly spatial patterns, in Fig. 3 we show the temporal evolution of the z-score anomaly as a function of consolidation stage during the onset of the late 2010 drought for a spatial window roughly covering the Horn of Africa region. For better readability, we show only three consolidation stages (the first available, C0; an intermediate one, C2; and the final fully smoothed one CF) of the N*x*H*x* computation option. The actual impact of the drought on vegetation development is well documented by the CF anomaly showing a progressively deteriorating situation (for a chronicle of the warnings and alerts issued by several international organizations see Hillbruner and Moloney, 2012).

Although the general picture depicted by the evolution of the CF anomaly (zNfHf, Fig. 3G–I) is captured by both C0 (zN0H0, Fig. 3A–C) and C2 (zN2H2, Fig. 3D–F), both NRT products underestimate the severity of the droughts. Qualitatively, C0 appears to lag behind CF (cf. Fig. 3B with G and C with H) with detrimental effect on the timeliness in spotting that a major problem was shaping up. In particular, at the earliest stage (dekad 27), C0 shows an overestimate of the area occupied by positive anomalies in the south-east of Ethiopia and north of Somalia and an underestimate of the negative anomalies in south Somalia (cf. Fig. 3A with 3G). Later on (dekad 33), when the effect of the drought is manifest, C0 (Fig. 3C) does not differ substantially from CF (Fig. 3I). This is however suboptimal for drought detection, where capturing early signals of deterioration is a fundamental component of the warning function.

**Fig. 4:**
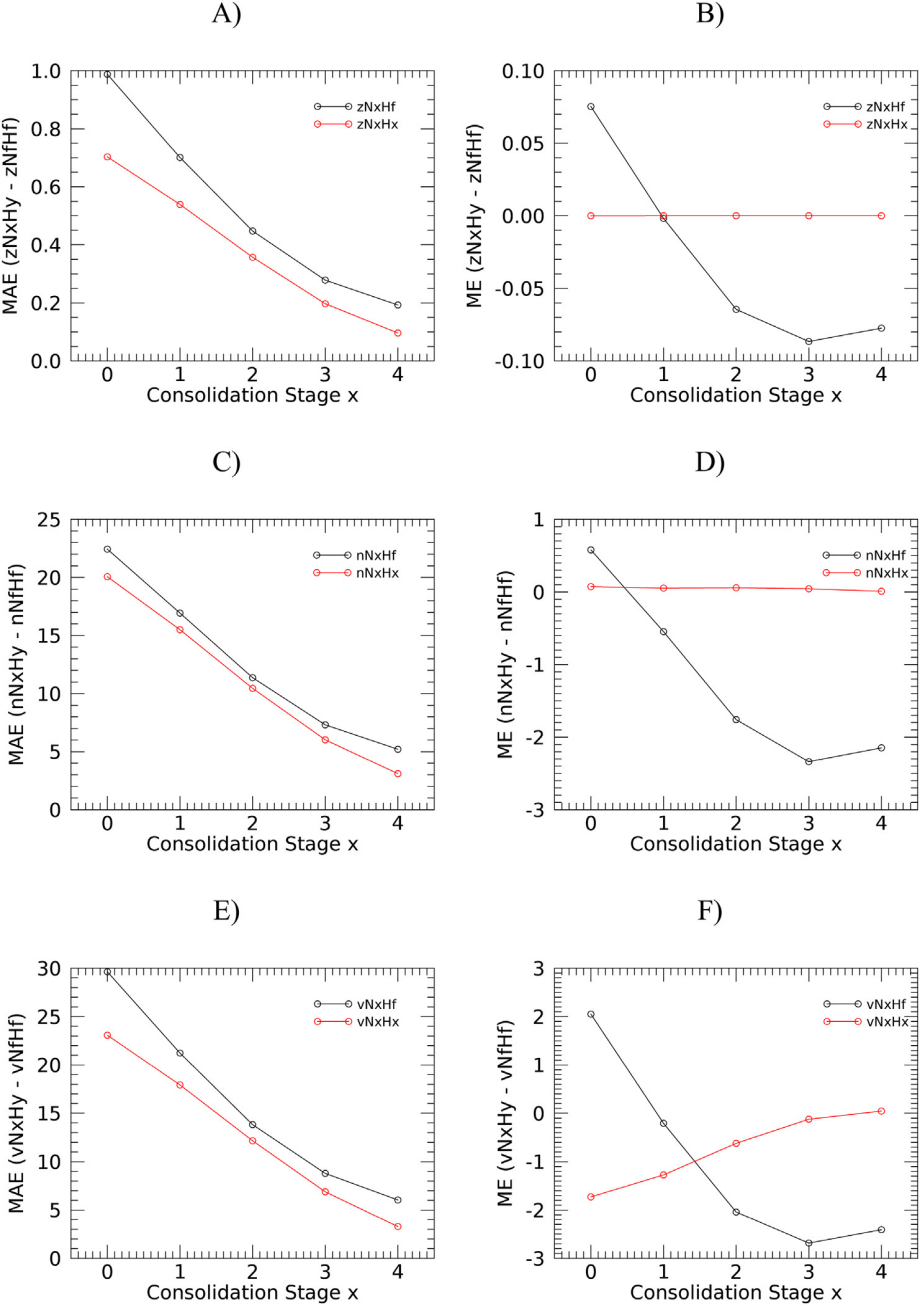
MAE (left panels), mean absolute value of the difference between NRT and reference anomalies (*a*NxHy-*a*NfHf) and ME (right panels), mean difference. MAE and ME are plotted as a function of consolidation stage for three anomaly types: z-score (A and B), NEP (C and D), and VCI (E and F). Black lines are for the anomalies constructed using the same consolidation stage for the NRT and historical statistics (N*x*H*x*). Red lines are for the anomalies using the final consolidation stage for the historical statistics (N*x*Hf). (For interpretation of the references to colour in this figure legend, the reader is referred to the web version of this article.)

The global MAE and ME (computed over the archive of global images) of the NRT anomaly estimation by consolidation stage and anomaly computation option (N*x*Hf vs. N*x*H*x*) are presented in Fig. 4.

Errors are large for the first unconsolidated stages. For example, the error in NEP obtained with the first consolidation stage shows the estimated probability of non-exceedance is for N*x*H*x* on average 20 percentage points away from the reference probability.

Independently from the consolidation stage used for the computation of the long term statistics, the error decreases with the use of an increased NRT consolidation stage for all the anomaly types as the filtering yields more reliable outcomes. The magnitude of MAE using NRT consolidation stage C0 is four to five times higher than that using stage C4 (available with a 4 dekads delay). The MAE graphics of Fig. 4 well depict the trade-off between timeliness and accuracy and give clear indication that, whenever possible (i.e. in applications where timeliness is not of utmost importance), the use of higher consolidation stages is preferable. Temporal aggregation of different consolidation stages to compute monthly averages (e.g. Klisch and Atzberger, 2016) or averages over the growing season experienced so far (e.g. Rembold et al., 2018) appear effective strategies to reduce the errors associated with the most recent stages, but obviously lead to a different type of anomaly where the most recent information is blended with previous observations.

For all the anomaly types considered, those obtained using the same consolidation stage for the NRT and the computation of the statistics (*a*N*x*H*x*) show a smaller MAE than those obtained using the final stage for the statistics (*a*N*x*Hf). In addition, scaling the NRT observation with the statistics derived from its distribution (*a*N*x*H*x*) results in unbiased estimation of the z-score and NEP anomalies (Fig. 4, right panels). This is not the case for initial stages of VCI where underestimation occurs for N*x*H*x* due to large errors in the estimation of distribution extrema, likely due to outliers (abnormally high or low NDVI values) present in the historical distribution of initial consolidation stages. Nevertheless, the bias of vN*x*H*x* is lower than that of vN*x*Hf in all stages except C1.

The value of ME of N*x*Hf anomalies with increasing consolidation stage is in all cases ranging from (slightly) positive to negative, suggesting that the filtering procedure appears to overestimate initial stages and underestimate the final ones. In summary, anomalies obtained using the same consolidation stage for the NRT and long term statistics have smaller MAE and bias (nearly no bias in the case of z-score and NEP). This finding suggests that this type of computation option should be used when averaging the anomalies over space, for instance when computing administrative level statistics.

**Fig. 5:**
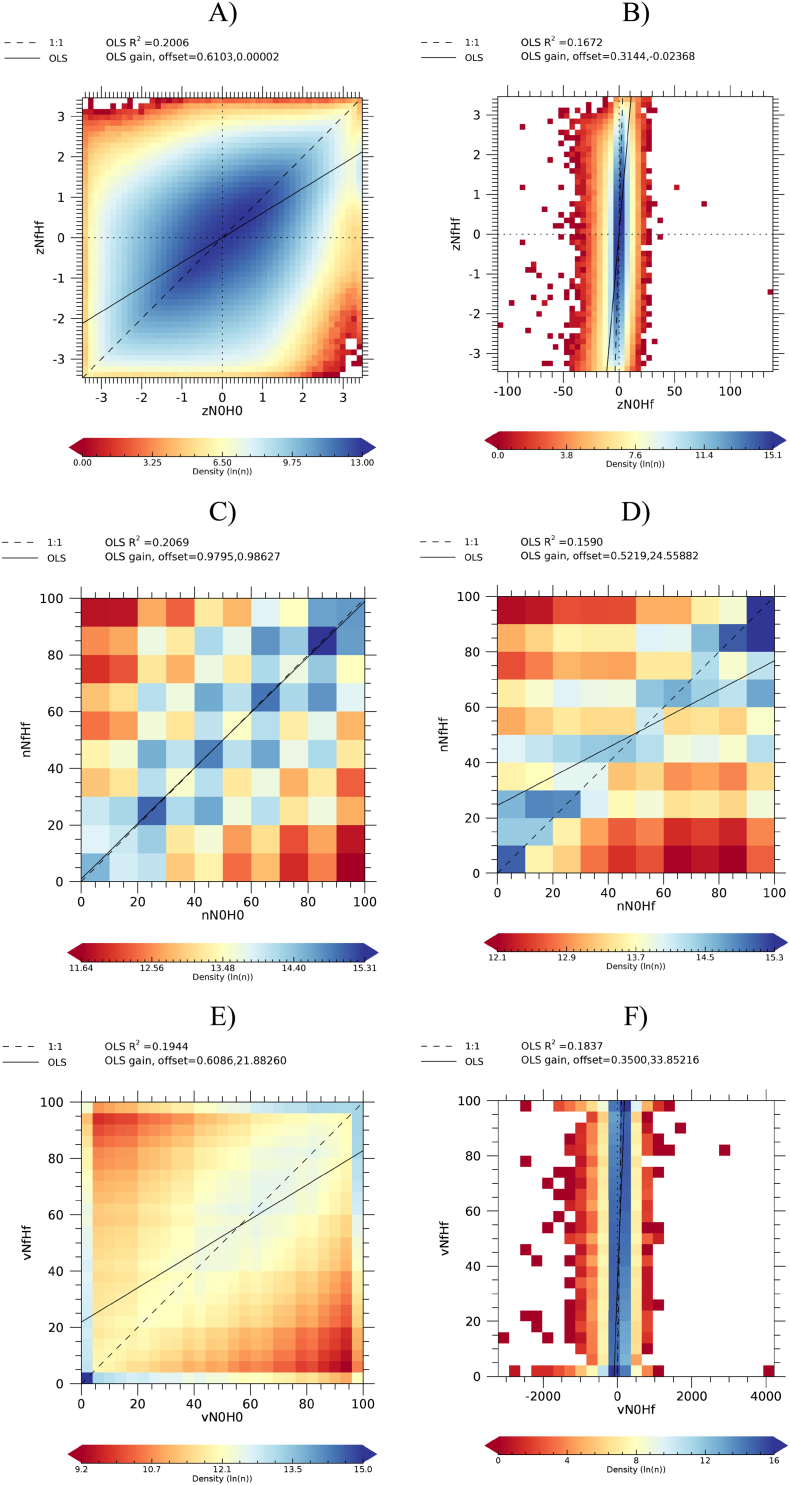
Density scatter plot *a*N0H0 vs. *a*NfHf (left panels) and *a*N0Hf vs. *a*NfHf (right panels). A) and B): z-score; C) and D): NEP; E) and F): VCI.

Focusing on anomalies computed for NRT data at consolidation stage C0, Fig. 5 shows the density scatter plots *a*N0H0 vs. *a*NfHf and *a*N0Hf vs. *a*NfHf, for the three anomaly types.

The variability of the *a*NfHf anomalies is better explained by the *a*N*x*H*x* anomalies compared to the *a*N*x*Hf ones (larger R^2^ for *a*N*x*H*x* in all cases, thus including *a*N0H0 to *a*N4H4). However, the fraction of variability explained is small (about 20% for *a*N0H0). R^2^ values increase with increasing consolidation stages (data not shown) and reach values larger than 0.5 for the last stage C4. With exception of nN0H0, the variability of the estimated anomalies is always greater than that of the reference anomaly. This is shown by the slope of the OLS regression that is in all cases significantly lower than 1. For z-score and VCI anomalies, it is interesting to note that *a*N*x*H*x* anomalies preserve the range of the reference anomaly whereas the range of *a*N*x*Hf anomalies is expanded as a consequence of the type of normalization. Interestingly, VCI can get values far outside its meaningful [0, 100] range. It is noted that truncating the VCI values to its physical range is possible but only marginally changes OLS statistics (data not shown).

### 4.2 Analysis of dichotomous and multi-category anomaly classification

**Fig. 6:**
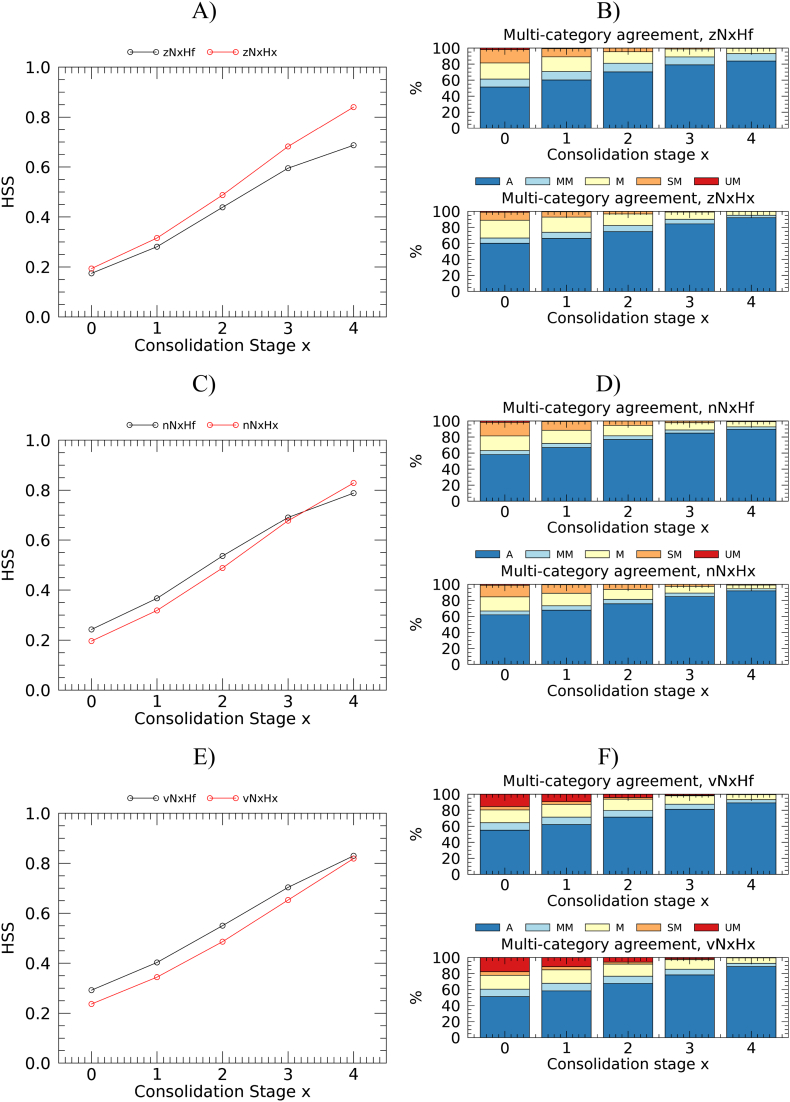
Verification metrics for multi-category anomaly classification. Heidke skill score (left panels) and multi-category agreement (right panels) as a function of consolidation stage. A) and B): z-score; C) and D): NEP; E) and F): VCI.

**Fig. 7:**
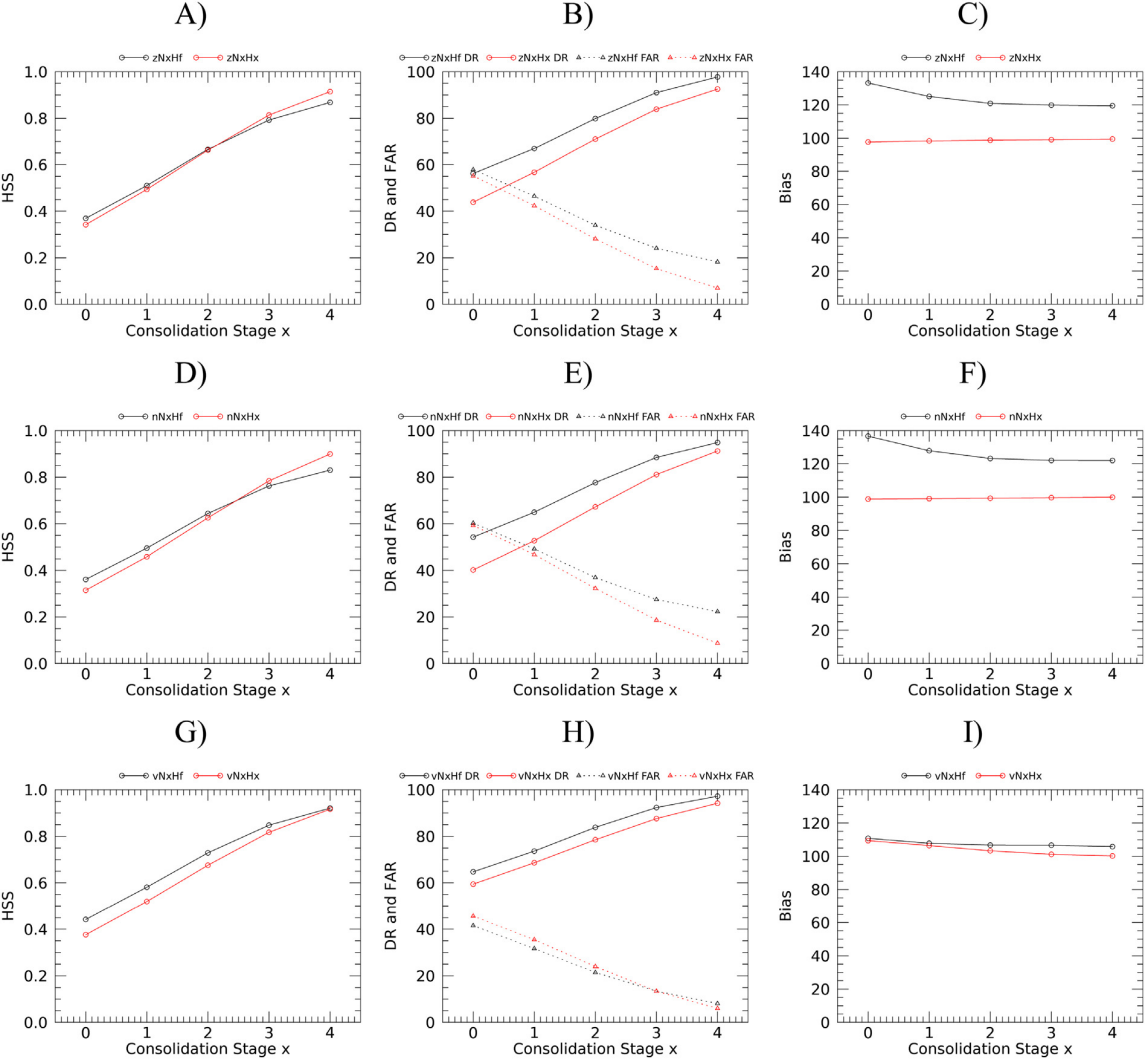
Verification metrics for dichotomous anomaly classification. Heidke skill score (left panels), detection and false alarm rate (central panels), and bias (right panels) as a function of consolidation stage. A), B) and C): z-score; D), E) and F): NEP; G), H) and I): VCI.

Fig. 6, Fig. 7 show the statistics of the multi-category and dichotomous anomaly classification agreement as a function of consolidation stage.

Results of the multi-category classification (Fig. 6) show that, when looking at the typical classification accuracy metric (HSS), the N*x*Hf option slightly outperforms the N*x*H*x* one for NEP and VCI. The contrary is true for the z-score. However, the HSS metric equally penalizes any misclassification type (e.g. a misclassification between “extremely bad” and “extremely good” conditions counts as one between “very bad” and “extremely bad” conditions). The multi-category agreement instead classifies the various misclassifications on the basis of the severity of the mismatch between classes. Looking at the sum of agreement and minor mismatch categories of the agreement plots, the N*x*Hf strategy is preferable only for VCI. In addition, it is only for VCI that the class of agreement “unacceptable mismatch” is significantly represented.

Results of the dichotomous classification at the first consolidation stage show poor to moderate agreement (Fig. 7, HSS from slightly below to slightly above 0.4). Lower detection rates, and higher false alarm rates are found for z-score and NEP compared to VCI. Detection rates are higher for *a*N*x*Hf anomalies that all show a positive bias. On the contrary, *a*N*x*H*x* anomalies have no bias with the exception of VCI (for which the bias is moderate for both computation options). All statistics improve nearly linearly with consolidation stages, and the agreement turns from moderate to strong (HSS > 0.8) at consolidation stages 3 or 4. With the typical pragmatic objective of maximizing detection rate and minimizing the false alarm rate, the best option for anomaly computation appears be the N*x*Hf, having a higher detection rate in all cases, and lower (VCI) or only slightly higher false alarm rate (z-score and NEP) at initial consolidation stages. Detection rates differences between the two anomaly computation strategy can be large, especially in the first stages. For example, nN0H0 and nN0Hf have a detection rate of 40 and 54%, respectively.

### 4.3 Spatial patterns

**Fig. 8:**
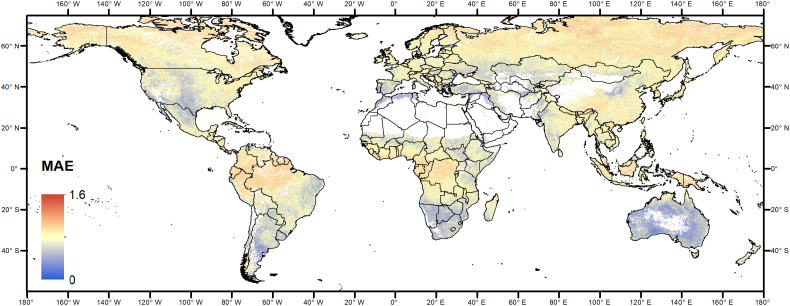
Map of the mean absolute value of the difference zN0H0 − zNfHf, for vegetated pixels during the growing season.

**Fig. 9:**
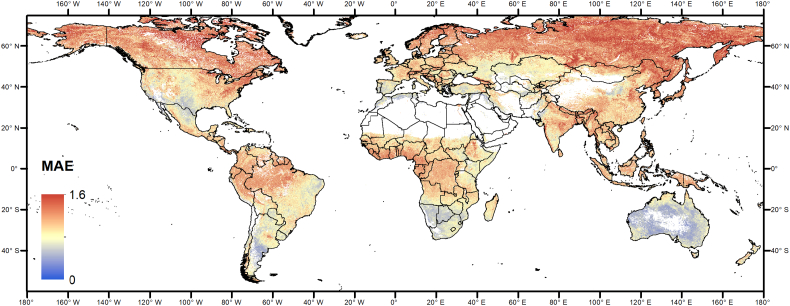
Map of the mean absolute value of the difference zN0Hf − zNfHf, for vegetated pixels during the growing season.

As an example, Figs. 8 and 9 show the global maps of the mean absolute error of zN0H0 and zN0Hf, respectively. Spatial pattern of the others anomaly types (NEP, VCI; data not shown) are similar.

The MAE of the two types of anomaly computation options has similar spatial patterns, MAE differs only in magnitude, with error of zN0Hf being larger as discussed in Section 4.1. We also observe that large variability in error magnitude is present, indicating that the reliability of NRT anomaly estimates is spatially variable. Prior knowledge about the mean estimation error as produced in this study may thus provide additional information for the interpretation of NRT anomalies in specific geographical areas. Where estimation error are large for the initial estimation stages, anomaly maps should be thus evaluated with care, without over-interpretation of situations only moderately above or below normal.

**Fig. 10:**
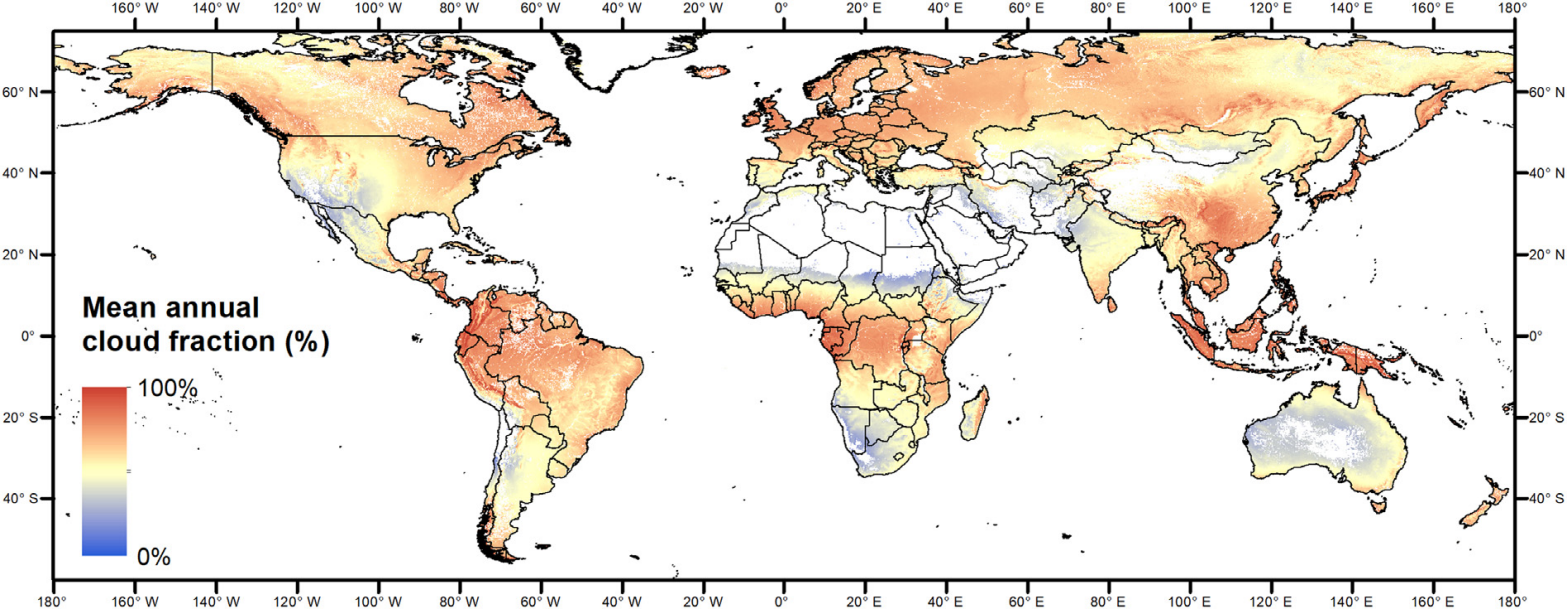
Mean annual cloud fraction over land (Wilson and Jetz, 2016).

The larger errors are located in the northern and high latitude areas and around the equator. As a possible explanatory variable for the anomaly estimation error, Fig. 10 shows the mean annual cloud cover fraction. Interestingly, the overall pattern is very similar, confirming that cloudiness is a major driver of the quality of EO-based indicators of vegetation anomalies.

**Fig. 11:**
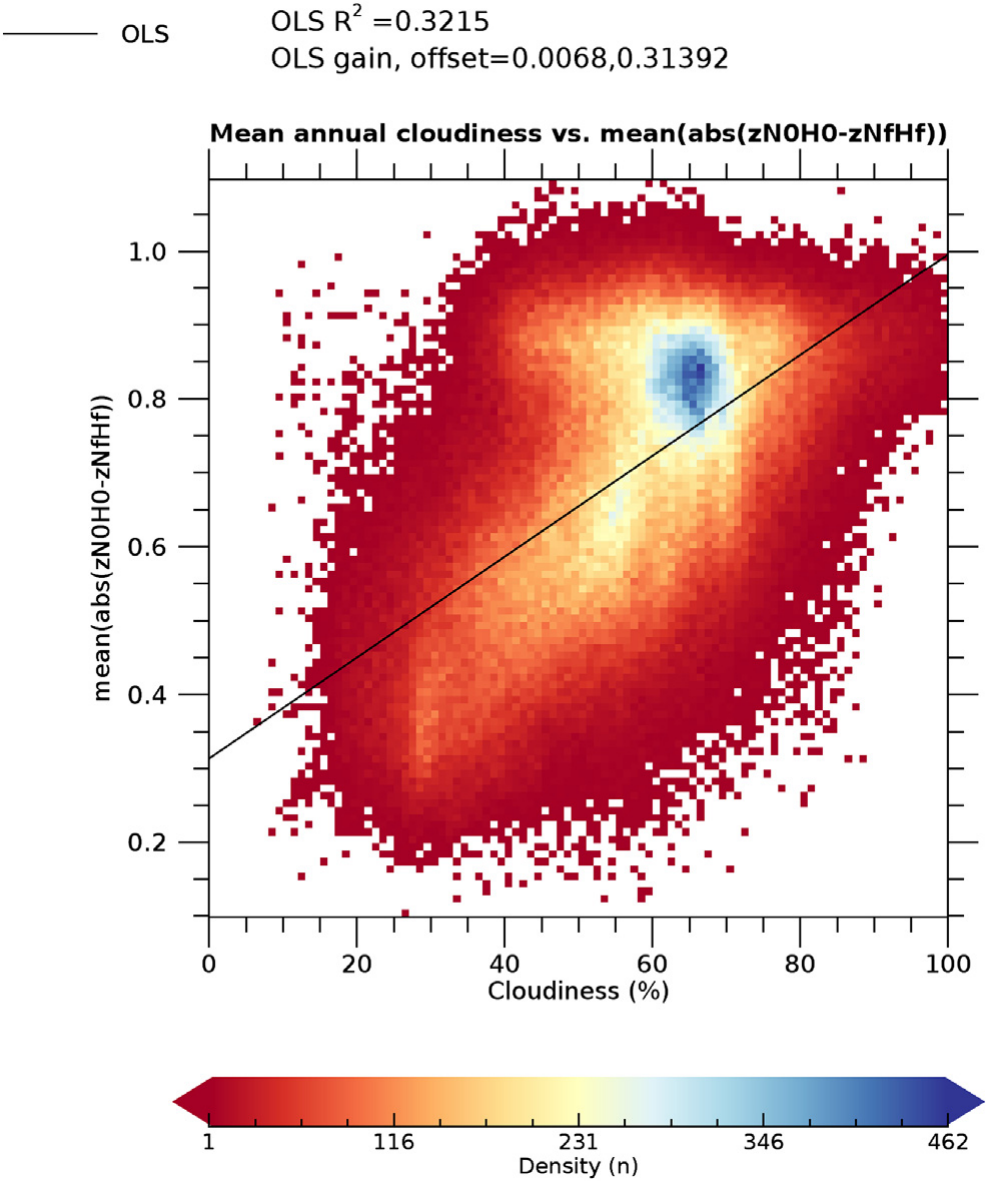
Density scatter plot of mean annual cloudiness vs. MAE of zN0H0 (data of Fig. 7, Fig. 9). The black line represents the ordinary least square (OLS) linear regression line.

Cloudiness explains a significant part of the variability of MAE (32% for zN0H0 as an example, Fig. 11). Additional factors explaining the errors at high northern latitudes may be related to snow cover and reduced incident radiation at time of satellite overpass (Jönsson et al., 2010), both resulting in gaps in the NDVI time series but not specifically addressed here.

## 5 Conclusion

Timeliness and accuracy are essential data characteristics for near real-time monitoring of vegetation. For the first time we focused on NDVI anomaly indicators, where small errors in the original NDVI data may be amplified by the anomaly computation. In this framework, we assessed the effect of different anomaly computation options on accuracy and the trade-off between timeliness and accuracy. The fully consolidated NDVI time series (off-line smoothing) was used as reference for our accuracy calculations. Results of this study provide practical information and guidance to both producers and users of NRT filtered products.

We showed that near real-time anomaly information at initial consolidation stages are affected by relatively large estimation errors. Additional effort in refining NRT filtering algorithms resulting in substantial improvements of the quality of NRT product is advocated. For example, the use of alternative sources of NDVI data could be an option for improving the quality of NRT filtered products. In particular, the NASA Land, Atmosphere Near real-time Capability for EOS (LANCE) system provides NRT MODIS data with reduced latency compared to NASA LP DAAC used by the filtering algorithm of this study. This data source would thus increase the availability of recent observations for the NRT filtering.

As expected, we found that initial estimation error is reduced when more satellite observations become available. As a general indication to user, results show that the quality of the anomaly is roughly doubled after two time steps (i.e. at consolidation stage 2). Analyst's confidence in the detection of an emerging problem can thus build up with subsequent updates as the new information confirms the old one, which at the same time becomes more reliable.

Results show that, depending on user's application, informed choice of anomaly type and computation option ensures the highest quality. For example, we found that anomaly estimates obtained using the same consolidation stage for the near real-time and long term statistics (N*x*H*x*) are more accurate than those obtained using the fully consolidated stage for extracting the statistics. The smaller MAE and bias indicate that this type of computation should be used if the final purpose is to compute average anomalies over geographical areas (e.g. administrative units). When the objective is to spot the occurrence of poor vegetation growth (represented by anomaly values located in the lower part of the observed distribution), the N*x*Hf computation strategy applied to VCI anomaly provided the highest accuracy and sensitivity.

When the objective is to reclassify the anomaly maps into few classes (separating normal, above or below normal, and extreme conditions) results show that z-score and NEP anomalies present the best agreement between estimated and final classes in terms of ranked concordance (i.e. penalizing those estimation errors that would indicate a condition contrasting with the reference one, for instance estimated extremely bad vs. reference extremely good). For these anomalies, the N*x*H*x* strategy outperforms N*x*Hf in terms of ranked concordance.

Finally, the accuracy metrics employed in this study for the different application scenarios may be adopted by producer of filtered NRT remote sensing products to characterize the quality of their data and allow intercomparison among products.

## Footnotes

1 E.g. the Famine Early Warning Systems Network (FEWSNET) of US Agency for International Development; the Vulnerability Assessment and Mapping Unit of World Food Program (WFP VAM); the Global Information and Early Warning System of the Food and Agriculture Organization (FAO GIEWS); the Global Agricultural Geo-monitoring Initiative of the Group on Earth observation (GEOGLAM); the Food Security Unit of the Joint Research Centre (JRC).
